# “OUR NATION’S DEMOCRACY IS AT A CRITICAL CROSSROAD”: OLDER ADULTS TAKING ACTION AND LEADING COMMUNITY ENGAGEMENT

**DOI:** 10.1093/geroni/igad104.1510

**Published:** 2023-12-21

**Authors:** Tam Perry, Vanessa Rorai, Sarah Whitney, Wilma Stringer, Henry Swift, James Bridgforth, Patricia Mullin

**Affiliations:** Wayne State University, Detroit, Michigan, United States; Healthier Black Elders Center, Detroit, Michigan, United States; Healthier Black Elders Center, Detroit, Michigan, United States; Healthier Black Elders Center, Detroit, Michigan, United States; Healthier Black Elders Center, Detroit, Michigan, United States; Healthier Black Elders Center, Detroit, Michigan, United States; Healthier Black Elders Center, Detroit, Michigan, United States

## Abstract

Launched by older Black adults from the Healthier Black Elders Center (HBEC) of the Michigan Center for Urban African American Aging Research, Critical Crossroads was created to engage older Detroiters in community conversations, advocacy, and strategies around issues of social injustice. Critical Crossroads provides older adults a space to discuss what concerns them at both national and local community levels, share their personal impact stories and experiences, and learn how these issues connect to social determinants of health. One important feature of this program has been attention to concerns of housing and its impact on older adults. This presentation will highlight two Critical Crossroads housing related segments (e.g., CDC moratorium on eviction, applying for FEMA aid for flooding). This presentation will share the This presentation will share the development of this community-led initiative, program goals, and lessons learned.

